# Unraveling the Risk Factors and Etiology of the Canine Oral Mucosal Melanoma: Results of an Epidemiological Questionnaire, Oral Microbiome Analysis and Investigation of Papillomavirus Infection

**DOI:** 10.3390/cancers14143397

**Published:** 2022-07-13

**Authors:** Joyce Pires de Carvalho, Marcella Collaneri Carrilho, Denner Santos dos Anjos, Carolina Dagli Hernandez, Laura Sichero, Maria Lúcia Zaidan Dagli

**Affiliations:** 1 School of Veterinary Medicine and Animal Science, University of Sao Paulo, Sao Paulo 05508-270, SP, Brazil; joycarvalho@usp.br (J.P.d.C.); marcella.carrilho@usp.br (M.C.C.); 2 Eletro-Onkovet Service, Franca 14406-005, SP, Brazil; denner.anjosoncology@gmail.com; 3 School of Pharmaceutical Sciences, University of Sao Paulo, Sao Paulo 05508-000, SP, Brazil; caroldagli@hotmail.com; 4 Center for Translational Research in Oncology, Instituto do Câncer do Estado de São Paulo-ICESP, Hospital das Clínicas da Faculdade de Medicina da Universidade de São Paulo FMUSP HC, Sao Paulo 05403-000, SP, Brazil; lsichero@gmail.com

**Keywords:** melanoma, dogs, microbiome

## Abstract

**Simple Summary:**

Oral mucosal melanoma (OMM) is one of the most common oral cancers in dogs; however, the risk factors for its development remain obscure and the etiology is unknown. This study aimed to investigate the risk factors and etiology of OMM in dogs. An epidemiological questionnaire was applied to the owners of 15 dogs with OMM and their paired controls, and the oral microbiome was comparatively determined in the two groups. Additionally, the presence of papillomavirus was investigated in the same OMM samples. Most OMM and control dogs had grade 3 periodontal disease. No risk factors were identified through the epidemiological questionnaire, and papillomaviruses were not identified in the samples. The bacteria *Tannerella forsythia* and *Porphyromonas gingivalis* were significantly overrepresented in dogs with OMM, and their presence could be considered a risk factor for the development of canine OMM.

**Abstract:**

Oral mucosal melanoma (OMM) is the most common oral cancer in dogs and is very aggressive in this species; its risk factors and etiology are yet to be determined. This study aimed to unravel the risk factors for the development of OMM in dogs and to investigate the possible presence of papillomaviruses as an etiological factor. A case-control study was conducted in 15 dogs with OMM and 15 paired controls whose owners answered an epidemiological questionnaire. Oral swabs from the same dogs were subjected to 16S rRNA sequencing for microbiome analyses. In addition, DNA fragments of OMM had their DNA extracted and amplified by polymerase chain reaction in an attempt to detect canine papillomaviruses. The gingiva was the most frequent anatomical site (47%) of OMM, and most tumors were stage III when diagnosed. Most dogs bearing OMM and the controls had grade 3 periodontal disease, and this factor, along with tartar treatment and tooth brushing, did not differ between cases and controls. Most dogs with OMM and most controls had contact with smokers; there was no statistically significant difference. Canine papillomaviruses were not detected among OMM cases. *Tannerella forsythia* and *Porphyromonas gingivalis* were significantly increased in case dogs compared to the controls. As these bacteria are reportedly involved in the pathogenesis of periodontal disease and esophageal cancer in humans, we suggest that they might be risk factors for the development of canine OMM. The limitations of this study include the low number of dogs, and therefore, further studies on canine OMM with larger numbers of animals are encouraged.

## 1. Introduction

Oral mucosal melanomas (OMM) originate from melanocytes within the mucosal epithelia. They can undergo neoplastic transformation, grow locally, invade adjacent tissues, migrate to distant organs, and generate metastases [1]. Although global data on canine cancers are not available, many retrospective studies have shown that OMM are prevalent neoplasms in the oral cavity [2,3,4,5,6,7]. They accounted for 0.99% of all canine neoplasms diagnosed at the University of Missouri [2]. In a study at the University of São Paulo (USP), 3% of 1813 cases of neoplasms in dogs were melanocytic [6]. Another study at USP included 2154 neoplasms between 2000 and 2006 in all species, and 8.9% of them were melanocytic; 186 cases occurred in dogs (96.4%) [7]. Male, mixed-breed dogs with black coats, from 8 to 11 years of age, are the most commonly affected animals [6]. In a survey of 384 melanocytic canine tumors, 19% were OMM [8].

According to the Global Cancer Observatory, GLOBOCAN, the estimated number of new cases of all cancers in humans, excluding non-melanoma skin cancer in 2020, was 18,094,716, and the estimated number of new cases of cancer in the lips and oral cavity in 2020 was 377,713 [9]. Pigmented melanocytic lesions are considered uncommon in the human oral mucosa, representing 0.8–3.7% of all melanomas [10]. These lesions include oral and labial melanotic macules, oral melanocytic nevi, oral melanoacanthomas, oral melanomas, and atypical melanocytic proliferations [11]. A retrospective study of oral pigmented neoplasms in humans showed that among 77,074 lesions diagnosed in this period, 761 (0.99%) represented pigmented lesions of the oral mucosa, including 351 (46.1%) melanocytic and 410 (53.9%) non-melanocytic lesions [12]. OMM is a highly aggressive tumor with poor prognosis in humans [1,10].

Despite the fact that OMM is common in dogs and rare in humans, the risk factors and etiology in both species are yet to be determined. Cancer risk factors increase the risk of neoplastic transformation. Some chemical and physical agents, hormones, and viruses are known carcinogens in humans and can also be considered as such in companion animals [13]. Some risk factors for human cancer include age, family history, use of tobacco products, radiation or certain chemicals, infections with certain viruses or bacteria, and genetic changes. Exposure to ultraviolet radiation is a risk factor for exposed areas of the body, such as the skin, but not a risk factor for OMM. Feller et al. [1] reported that in humans, the only known risk factor for OMM is the presence of hyperpigmented oral mucosa. In a recent systematic review, Thuaire et al. [14] described that probable risk factors for human OMM include chronic mechanical trauma, alcohol and tobacco consumption, and exposure to formaldehyde. A possible genetic predisposition has been hypothesized for canine OMM, due to the heavily pigmented oral mucous membranes in some breeds [3].

The clinical, pathological, and prognostic features of canine melanoma have recently been reviewed [15,16]. OMM can be classified as melanotic or amelanotic, and its amelanotic counterpart is considered more aggressive than its melanotic counterpart [17,18]. Spontaneous melanomas in dogs, including OMM, are considered good models of human melanomas [19,20,21]. In addition, dogs are sentinels of environmental contamination to which humans are exposed [22] and are good translational models [23].

Well-conducted epidemiological studies may help elucidate the factors or agents that can contribute to pathological conditions. Observational studies are epidemiological tools used to identify and characterize the determinants of cancer risk [24]. Case-control studies are epidemiological and observational studies in which the subjects are selected according to their pathological condition and observed to determine the factors that contribute to their outcomes. In this study, we performed a case-control study on dogs bearing OMM in comparison with controls in an attempt to identify possible contributing factors. For this purpose, OMM patients and controls were included in the study, and their owners answered an epidemiological questionnaire. Epidemiological questionnaires are considered suitable tools for assessing exposure and are frequently used in exposure assessments of occupational and environmental epidemiological studies. They can be used in combination with other methods to assess exposure and risk factors. According to Nieuwenhuijsen, very few, if any, standardized and validated questionnaires are available in this area [25].

Recently, Hanahan in 2021 [26] included the microbiome as an emerging hallmark and enabling characteristic for cancer development. The microbiome is composed of bacteria, archaea, fungi, protozoa, viruses, and their genomes, which are found on and within human and animal bodies. The microbiome is important for homeostasis, metabolism, gut epithelial health, immunological activity, and neurodevelopment. It also plays a fundamental role in the regulation of human health and diseases [27]. Therefore, a mechanistic understanding of complex microbiome–host interactions is crucial in several fields of research. Metagenomic sequencing is a powerful technology that aims to obtain all the genetic information contained in a given biological sample.

The canine oral microbiome has been focused on in two recent studies. Dewhirst et al. (2012) [28] analyzed 5.958 16S rRNA gene sequences from 65 clone libraries and verified that the bacterial content in dog mouths differs from that in human mouths, and only 16.4% are shared bacterial types in the two species. Ruparell et al. (2020) [29] compared the microbiota from different areas within the canine oral cavity and found three niches in which the bacterial community profiles differed: soft tissue surfaces (buccal and tongue dorsum mucosa), hard tissue surface (supragingival plaque), and saliva. To our knowledge, the microbiome of the oral cavity of dogs with OMM has not yet been investigated.

Papillomaviruses are found in many animal species, mostly causing epithelial neoplasms. In veterinary medicine, there is evidence connecting canine papillomavirus with oral/skin squamous cell carcinoma in dogs. Papillomaviruses are associated with viral plaques and feline fibropapillomas in cats. The mechanism of the transformation of benign neoplasms into squamous cell carcinoma remains unclear [30,31]. In this study, we investigated whether papillomavirus is involved in the development of OMM in dogs.

Here, we investigated the epidemiological aspects of canine OMM through a case-control study, the oral microbiome through 16S rRNA sequencing, and the expression of papillomaviruses in OMM cases to elucidate the possible risk factors and etiology of OMM.

## 2. Material and Methods

### 2.1. Ethics

This study was approved by the Committee of Ethics on the Use of Animals (CEUA) of the School of Veterinary Medicine and Animal Science at the University of São Paulo (USP) (process number 8614150218). Dog owners signed an informed consent form.

### 2.2. Animals and OMM Samples

Fifteen dogs with OMM were selected from among patients seen at the Veterinary Hospital of the National Association of Clinicians (Anclivepa) Veterinary Hospital in the city of São Paulo, SP, Brazil. Canine OMM was diagnosed based on gross examination of the oral cavity accompanied by cytology or histopathologic incisional biopsy. The dogs underwent physical examination and other pre-surgical examinations, such as complete blood counts and a biochemistry panel. Subsequently, the patients were referred for proper treatment according to the owners’ decisions. The inclusion criteria included the presence of OMM, and patients who had previously received antibiotics were excluded.

The dogs underwent physical and other pre surgical examinations. All patients were staged based on the tumor, node, metastasis staging system proposed by the World Health Organization [32,33] including tumor diameter, lymph node involvement, and distant metastasis.

The procedure to obtain oral swab samples was standardized and consisted of rubbing the swab on the patient’s oral mucosa on the day of surgery, during anesthetic induction.

Periodontal diseases were evaluated by inspecting oral and periodontal spaces. A visual dental scale according to Bauer et al. [34] was used, and the periodontal disease was classified as 0 to IV.

After the surgical procedure, conducted in accordance with the protocols of animal welfare, the tumor samples were cut into 1 cm slices and conditioned in universal collectors with 10% commercial formalin solution. After fixation, the slices were routinely processed for inclusion in the paraffin blocks. The 5 μm thick sections were stained with hematoxylin and eosin for histopathological analysis and to confirm the diagnosis of OMM.

After the case group was obtained, 15 clinically healthy dogs free of any neoplasm were identified, including only patients of the same breed, sex, and age of the animals in the OMM group. Recruitment was performed at the Teaching Hospital of the School of Veterinary Medicine and Animal Science of the USP. The animals had heart or orthopedic disease. Saliva samples were collected from all dogs by scrubbing using oral swabs as long as they were waiting for non-hospital medical care.

### 2.3. Application of the Epidemiological Questionnaire

This study used a four-page computerized questionnaire available on the online platform Google Forms.

The epidemiological questionnaire consisted of 43 items, including owner data (name, telephone, address), animal data (name, age, breed, sex), presence or absence of oral melanoma, animal’s water and food regime, weight, body condition score, facial conformation, environment and its particularities in which the dog lives (location of residence, rooms most frequented by the animal, floor), contact with specific materials and products (burnt wood, construction materials, herbicides, fungicides, insecticides, household products, incense, candles, fragrances, perfumed oils, shampoo with ectoparasiticide, external anti-parasite), presence or absence of electronic equipment (wireless, cordless telephone), presence or absence of contact with smokers and their frequency, frequency of tooth brushing performed on the animal, performance of dental cleaning and history of periodontal disease, and location and staging of the tumor. The questionnaire was adapted from a questionnaire created by our group and used by Zanini et al. [35].

The raw data were inserted into a spreadsheet and further analyzed to obtain responses from the cases and controls. The questionnaire was administered by Marcella Collaneri Carrilho between 2018 and 2020.

### 2.4. Polymerase Chain Reaction (PCR) Amplification of Taupapillomaviruses

The presence of the canine papillomavirus (CPV) DNA was investigated by PCR. using primers designed to amplify fragments of the *L1* or *E1* genes of several canine papillomavirus. Primers were synthesized by Thermo Fisher Scientific (Wilmington, DE, USA) and are described in Table 1.

DNA from all tumor samples was extracted at the Molecular Biology Laboratory of the Cancer Institute of the State of São Paulo (ICESP) using a protocol with phenol, chloroform, and isoamyl alcohol; and the quality of the extracted DNA was evaluated by spectrophotometry using NanoDrop^®^ equipment (Thermo Scientific, Wilmington, DE, USA). All DNA was used in a PCR reaction using the appropriate primers capable of amplifying a fragment of the late *L1* gene. PCR was performed using the AmpliTaq Gold^®^ polymerase enzyme (Applied Biosystems, Foster City, CA, USA). A tissue-free negative control was included in all procedures to monitor for possible contamination.

The generated PCR products were purified using the Illustra ExoProStar 1-Step (GE Healthcare, Buckinghamshire, UK), and then the BigDye^®^ Terminator v3.1 Cycle Sequencing kit (Applied Biosystems, Foster City, CA, USA) was used for sequencing on an ABI 3130XL Genetic Analyzer automatic sequencer.

All PCR products were analyzed using 2% agarose gel electrophoresis, stained with ethidium bromide, and visualized under ultraviolet light.

Samples that did not generate good-quality sequences by direct sequencing of the PCR products were cloned using the TOPO TA Cloning^®^ kit for sequencing (Invitrogen, Carlsbad, CA, USA), according to the manufacturer’s instructions. The generated colonies were grown, and the DNA was isolated by miniprep using the Wizard Plus SV Minipreps DNA Purification System (Promega, Madison, WI, USA) and then sequenced. The generated sequences were identified using the BLAST program (https://blast.ncbi.nlm.nih.gov/Blast.cgi (accessed on 3 July 2022)).

### 2.5. Analysis of the Microbiome by 16S RNA Sequencing

Dry sterile flexible swabs of flocculated nylon were used to obtain oral saliva from 30 animals. The swabs were placed in a microtube containing a buffer solution and then sent to the company Neoprospecta (https://www.neoprospecta.com/ (accessed on 3 July 2022), based in Florianópolis, SC, Brazil, in plastic packaging that was properly identified and individualized. The criteria for inclusion in the study were as follows: animals could not be under local or systemic antibiotic therapy in the last 30 days, did not eat food or water in the last two hours, and had not received any oral topical treatment.

Following the best sample collection practices, with proper storage and transport conditions to preserve bacterial genetic material, small amounts of DNA (less than 0.05 ng) were extracted. Sequencing library preparation was performed using a two-step PCR protocol, providing the best primer amplification and optimization for multiplexing.

In the first PCR reaction, the primers V3 and V4 341F-806R were used because of their high taxonomic coverage.

The protocol used primers for the V3/V4 regions with the following conditions: the first PCR primers contained the Illumina sequences based on the TruSeq structure (Illumina, San Diego, CA, USA), allowing the second PCR with indexing sequences. PCR reactions were performed in triplicate with the enzyme Platinum Taq (Invitrogen, Waltham, MA, USA) with the following conditions: 95 °C for 5 min; 25 cycles of 95 °C for 45 s, 55 °C for 30 s, and 72 °C for 45 s; and a final extension at 72 °C for 2 min for PCR 1.

For PCR 2, the conditions were 95 °C for 5 min; 10 cycles of 95 °C for 45 s, 66 °C for 30 s, and 72 °C for 45 s; and a final extension at 72 °C for 2 min. For comparison, the Illumina 16S rRNA protocol was used as described by Hoang et al. [36]. The final PCR was cleaned using AMPureXP beads (Beckman Coulter, Brea, CA, USA), and samples were pooled into sequencing libraries for quantification.

Estimates were performed with Picogreen dsDNA assays (Invitrogen, Waltham, MA, USA), and then pooled libraries were diluted for accurate qPCR quantification using the KAPA Library Quantitation Kit for Illumina Platforms (KAPA Biosystems, Woburn, MA, USA). Libraries were sequenced on a MiSeq system using the standard Illumina primers provided in the kit. A single-ended 300 nt run was performed.

After sequencing, the bioinformatics pipeline was used for sequence demultiplexing. The sizes of the reads were normalized to 283 bp. To increase read reliability, excluding the possible diversity generated by chimeric amplicons or erroneous nucleotides embedded in PCR, we pooled 100% identical reads. If any cluster was represented by less than five reads, it was not considered for further analysis.

Each cluster obtained a unique identifier, allowing traceability between results, which allowed us to compare operational taxonomic units (OTUs) from different experiments, taxonomies with different pipelines, and benchmark database analysis. Using this read-clustering approach, we established that the cluster is an OTU.

Hypervariable regions V3–V4 and V4 were amplified with primers 341F-806R and 515F-806R in triplicates of the same sample. The V4 region alone showed less resolution for classifying some species, such as *Staphylococcus aureus* and *Enterococcus faecalis*.

### 2.6. Statistical Analysis

In this study, numerical data were expressed as means or medians and compared using the Mann–Whitney test. Categorical data were expressed as frequencies and percentages and were compared between cohorts using Fisher’s exact test. All the variables of the individual characteristics of the dogs and the results of the 16 s rRNA sequencing were inserted into a Microsoft Excel spreadsheet, and the mean and standard deviation of these variables were calculated, to later perform the Mann–Whitney test to obtain the *p* value for each one. To be considered statistically significant, the p values of all variables should be equal to or less than 0.05. Graphs were constructed using GraphPad Prism software.

## 3. Results

### 3.1. OMM and Control Cases

All of the OMM in the 15 dogs were melanotic. Six animals were female and nine were male. Most animals were neutered and 7–10 years old. Mixed-breed (Mongrel) dogs were overrepresented in the OMM and control groups. Most dogs with OMM are mesocephalic in nature. Animals with periodontal disease were similarly represented in both the OMM and the control groups.

For the control group, 15 dogs with the same characteristics as the case group were recruited, as long as there was no previous diagnosis of any type of neoplasm. The population of dogs included in the case group consisted of nine male dogs and six female dogs.

These results are presented in Table 2.

### 3.2. Epidemiological Questionnaire Results

The results of the epidemiological questionnaire are summarized in Table 1, Table 2, Table 3 and Table 4.

Mixed-breed dogs represented 53% of the population and were the most prevalent (8 out of 15 dogs); 80% of the animals were neutered (12 of 15 dogs).

In both groups, 53% (8 out of 15 dogs) had periodontal disease, and only 7% of the owners practiced routine toothbrushing. There was no statistically significant difference in relation to periodic tooth brushing; it did not represent a protective factor against the disease.

An odds ratio (OR) calculation was applied to the samples, and none of the variables studied showed a significant correlation with canine oral melanoma.

The anatomical locations of the tumors were the gingiva (54%), upper lip (13%), hard palate (13%), soft palate (7%), lower lip (7%), and tongue (7%).

Seven dogs with OMM and eight controls had periodontal disease. All cases were classified as grade 3, according to Bauer et al. [34].

More than 93% of the animals in both groups received preventive commercial antiparasitic treatment at different time intervals. No statistically significant differences were observed in the use of this product. None of the evaluated variables related to the habits and handling of animals were identified as risk factors for canine oral melanoma.

### 3.3. Detection of Canine Papillomavirus in OMM Samples

Three DNA extractions were performed from fresh oral melanoma tumor samples from the 15 dogs in the case group at the Molecular Biology Laboratory of Cancer Institute of the State of São Paulo, ICESP. There was no successful amplification of canine papillomavirus DNA (CP4/CP5 and FAP64/CANPV f) in the samples.

### 3.4. Characteristics of the Oral Microbiomes of Dogs with OMM

**Figure 1:**
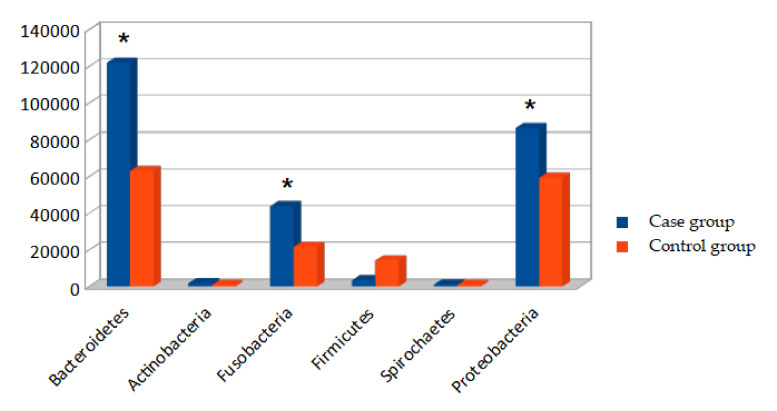
Bacterial phyla found in the oral cavities of dogs bearing OMM. * indicates that the values are significantly different.

Bacterial phyla found in the oral cavities of dogs bearing OMM are depicted in Figure 1.

Bacterial species whose numbers of sequences were less than 100 ng/dL in the samples were disregarded in the statistical analyses, given their low concentrations. The analyzed samples included 51 species and 7 different bacterial phyla.

The bacterial phyla found in greater abundance in both groups were Bacteroidetes, Proteobacteria, and Fusobacteria, which were found in significantly higher amounts in dogs in the case group.

The bacterial phyla found in smaller amounts were Synergistetes, Spirochaetes, and Actinobacteria, which were found in slightly higher amounts in dogs of the case group.

**Figure 2:**
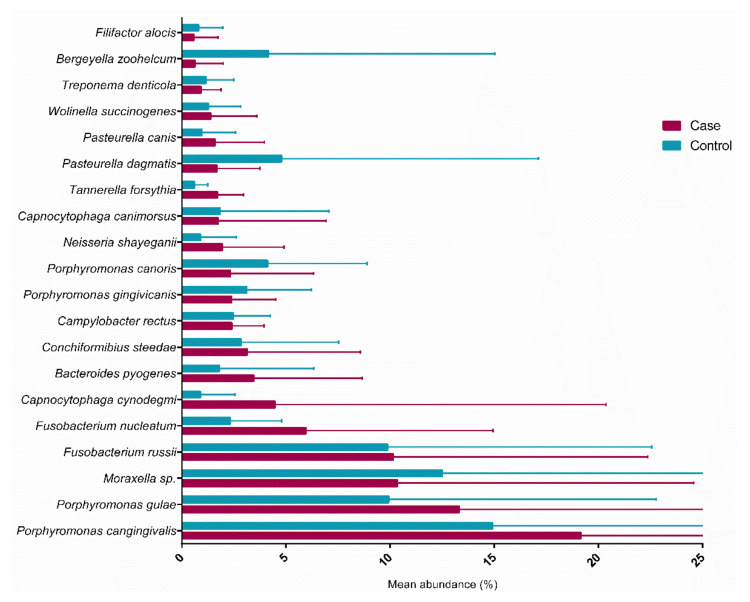
Bacterial species found in the oral cavities of dogs bearing OMM.

The most abundant bacteria in both groups were *Porphiromonas cangingivalis*, and they were found in significantly higher quantities (*p* = 0.017) in dogs in the OMM group compared to dogs in the control group. Literature relates the presence of this microorganism to the pathogenesis of periodontal disease. The bacterial phyla *Bergeyella zoohelcum* was significantly higher in control animals not bearing OMM. The significance of this finding is unknown. The least common bacterium in both groups was *Filifactor alocis*, and it was found in greater quantities in the animals of the case group. The bacterium *T. forsythia* was found in all samples of dogs with OMM and controls, though in significantly higher amounts in dogs with OMM (*p* = 0.017) (Table 5 and Figure 2). Its presence in the oral cavities of dogs with melanoma may be related to the etiopathology of this type of cancer.

## 4. Discussion

Canine cancers are important models of human cancers because they are spontaneous and share histological and behavioral similarities with their human counterparts [19,20,21]. In addition, because domesticated pet dogs in general share their environments with humans, they can be considered good sentinels for environmental contamination that may also affect their owners [22]. Since OMM in dogs and humans share important characteristics, such as morphology and behavior, and the risk factors and etiology are largely unknown in both species, as stated by Giuliano [23], we decided to investigate the etiological and risk factors of OMM to contribute to the control of this disease in dogs and humans.

In general, the precise etiology of specific neoplasms is difficult to identify, since numerous environmental or hereditary agents are known to contribute to carcinogenesis. Once evidence of a risk factor is obtained, primary prevention can be achieved by avoiding or minimizing these factors; therefore, the incidences of some cancers can be reduced. The literature suggests that the risk factors for canine and human OMM are similar and are related to chemical, microbiological, or physical agents [22].

This study aimed to unravel the risk factors for canine OMM. Three studies were conducted. In the first study, an epidemiological questionnaire was filled by the owners of dogs with OMM and controls. The second arm of the study referred to the evaluation of the oral microbiome, and in the third arm, we aimed to identify papillomaviruses in OMM samples.

Some risk factors can be identified for certain neoplasms, and epidemiological approaches have been used for this purpose. Epidemiological questionnaires are useful tools for identifying etiological and risk factors [24]. However, to the best of our knowledge, there are no validated questionnaires that can be used to identify environmental or occupational factors associated with cancer [25]. This was a limitation of the study, and we were extremely careful to avoid any bias in the exposure–disease association [24].

According to Nieuwenhuijsen [25], the design of new questionnaires often depends on the experience acquired from previous questionnaires. For this study, we adapted a questionnaire that had been used by our group to evaluate the risk factors for canine lymphoma [35]. The questionnaire (Supplementary Material) was well accepted and was administered to dog owners by the same person (Marcella Carrilho) to minimize eventual bias regarding the owners’ answers.

No differences were observed in the demographics, environmental factors, or habits of dogs with OMM compared with the paired controls.

The gingiva was the most frequent anatomical location where OMM was found in the dogs involved in this study (53% of the animals). Most of the OMM-bearing dogs and control dogs had grade 3 periodontal disease, evaluated using a visual scale proposed by Bauer et al. [34]. Periodontal/inflammatory disease and tartar have been considered risk factors for oral neoplasms in humans, as inflammation is considered an enabling hallmark of cancer [26,37,38] and can be considered as the underlying cause of the neoplasm. However, in our study, periodontal disease and dental tartar were similar between dogs with OMM and controls. One reason for this is that we studied the OMM cases. A possible new study to evaluate the role of periodontal disease and tartar’s influence on the development of OMM would be a cohort study, in which dogs with oral and dental diseases could be followed through their lives to adulthood or the elderly stage.

All animals in this study were elderly and presented dental tartar accumulation to a greater or lesser degree; therefore, the wide range of microorganisms found in all of them can be justified. Genetic and familial factors must also be considered in the carcinogenesis of canine oral melanoma, and a large number of animals in the samples, to ensure the reliability of the results. No differences were observed in the answers on tobacco exposure, type of food, or tooth brushing. The use of insecticides, barbecue smoke, house cleaning products, and Wi-Fi responses were similar in the OMM and control dogs.

The results of the analysis of the oral microbiomes of the 30 dogs evaluated in this study indicated that the most abundant bacteria in the canine oral cavity belonged to the phyla Bacteroidetes and Proteobacteria, in approximately 8% greater quantities than those found in the dogs in the control group. This corroborates the characterization of the canine oral microbiome by Dewhirst et al. in 2011 [28]. Dogs selected to comprise the OMM and control groups were not taking antibiotics, as this was an exclusion factor. It was not recorded if the dogs were taking other drugs that could interfere with the microbiome analysis, for example, NSAIDs or others, and this may be a limitation of this study.

Although the associations between some species of bacteria and oral cancer have already been established in humans, the complexity of the relationship between cancer and the oral microbiota remains unexplained and cannot be limited to the study of a single bacterium. Healy and Moran (2019) [39] presented the possible mechanisms by which the oral microbiome could be involved in the etiology of oral cancers, especially squamous cell carcinoma (SCC). The altered microbiome may play a role in the malignant progression of SCC via inflammation or other mediators, thereby contributing to the development of cancer. As melanocytes lie among the basal cells of the epithelium, it is feasible to propose that they are susceptible to the same environmental conditions as the epithelium. The existence of pigmented oral mucosa can justify the malignant transformation of melanocytes in animals and humans.

In fact, higher presence of these bacteria can be a consequence of intense periodontal disease and inflammation, or contamination by pus and blood promoted by cancer, or chronic infection in the oral cavities of these animals with periodontal disease prior to cancer.

The bacterium *Tannerella forsythia* belongs to the phylum Bacteroidetes and synthesizes serpin protein, which protects bacteria against the proteolytic effects of neutrophils. Epithelial adhesion and proliferation in the host are guaranteed by the S-glycoproteins in its surface layer and by the BspA proteins, which bind to extracellular fibronectin and fibrinogen. Glycosidases hydrolyze oligosaccharides and proteoglycans and provide nutrients to *T. forsythia*. In the presence of glucose, large amounts of methyl glyoxylic toxins accumulate, thereby promoting tissue damage. It also increases the population of *P. gingivalis* by reducing fumarate to succinate. *P. gingivalis*, owing to its high proteolytic activity, provides peptides and amino acids from T. forsythia, which are released from damaged tissues of the host [40].

There is evidence of the use of the oral microbiome in humans as a noninvasive tool for the diagnosis of gastrointestinal cancer, which has already been proven to be related to gastrointestinal cancer (colorectal, pancreatic, gastric, esophageal, and liver). Bacteria involved in periodontal disease, such as *P. gingivalis* and *T. forsythia*, are linked to several types of gastrointestinal cancer [40,41]. In one study, the population of oral bacteria differed significantly between patients with upper digestive cancer and healthy controls compared with colorectal cancer.

Changes in the oral microbiome are associated with the risk of developing gastrointestinal cancer. However, further studies are necessary to validate these findings. The studies must be based on large populations and present reproducible protocols for oral microbiome research, and descriptions of the transmission patterns of the oral gut microbiota must be resolved [40,41].

The importance of this study is the detailed description of the microbiomes in the oral cavities of dogs with oral melanoma, which has yielded pioneering results in this field of research with relevance for dogs and humans with OMM. The epidemiological questionnaire used in this study required a greater number of animals than usual to obtain more information. We also emphasize the need for more comparative studies related to the canine and human microbiomes, using follow-up of cancer patients during the development of the disease.

A limitation of this study was the small number of animals involved (15 in each group, for a total of 30 dogs). Zanini et al. (2013) [35] evaluated 80 dogs with lymphoma and 80 controls, and it was possible to significantly associate the occurrence of non-Hodgkin’s lymphomas with living close to busy avenues in the city of São Paulo. In contrast, De Holanda et al. [42] evaluated the environmental influences on the development of non-Hodgkin’s in dogs from the city of Recife, PE, Brazil. This case-control study included 89 dogs with lymphoma and 89 controls, and demonstrated that the occurrence of lymphomas is related to exposure to environmental carcinogens. Therefore, we encourage further studies on canine OMM, enrolling larger numbers of patients.

Another limitation of this study is that microbiome analysis has been performed in cases where the OMM has already been established. Therefore, it would be interesting to study the early phases of OMM development and the influence of the microbiome.

In 2000 and 2011, Hanahan and Weinberg [37,38] defined the characteristics of cancer as comprising six biological capabilities acquired during the development of various stages of human tumors. It has been established that inflammatory events that respond to tissue injury may favor tumor consequences of inflammatory responses. In the most recent version of the series, Hanahan (2021) [26], the authors reinforced the role of inflammation in the promotion of tumors and incorporated the characteristics of the microbiome as a factor that promotes tumor proliferation, adding to the characteristics already described.

## 5. Conclusions

In this study, *T. forsythia* and *P. cangingivalis* were found in significantly higher numbers in dogs with OMM. This may be a consequence of intense periodontal disease, inflammation, and contamination by pus and blood promoted by cancer, as they are chronically in the oral cavity of these animals, which is a periodontal disease prior to cancer. It is important to note that these are key bacteria in the development of periodontal disease in humans, and there are reports that they may contribute to the development of esophageal cancer.

This study aimed to unravel the etiology and risk factors of canine OMM. Epidemiological studies using questionnaires are suitable tools for the detection of cancer risk factors. Increasingly powerful experimental and computational technologies have provided a “big data” revolution for the myriad of diseases involved in the cancer process. Understanding that there are multiple causal factors helps us to better understand the mechanisms of cancer development and progression, which brings advances to medicine, both in cancer treatment and prevention.

## Figures and Tables

**Table 1:** Primers used to amplify the canine papillomavirus through PCR.

Primer Pair	Description
FAP64/CANPV f	Amplifies a fragment of CPV *L1* gene.
CP4 and CP5	Amplifies a fragment of CPV *E1* gene.
Dog GAPDH f and Dog GAPDH r	Amplifies a fragment of the canine GAPDH gene (used as internal control of DNA quality and sufficiency).

**Table 2:** Characteristics of the dogs included in the study and responses to the epidemiological questionnaire.

Dog	Breed	Sex	Neutered?	Age	Localization	Stage	Body Score	Periodontal Disease?	Smokers?	Brush the Teeth?	Tartar Cleaning?	Food Type	Eat Fruits and Vegetables?	Type of Housing	Contact with Pesticides?
OMM1	Mixed	F	Y	11	hard palate	III	obese	N	Y	N	Y	H	Y	house	N
OMM2	Cocker	F	Y	11	soft palate	II	adequate	Y	N	N	N	C	Y	house	N
OMM3	Poodle	F	N	11	gingiva	II	thin	N	N	Y	Y	C	N	house	N
OMM4	Mixed	F	Y	8	gingiva	III	adequate	Y	N	N	N	C	N	house	N
OMM5	Mixed	F	Y	13	tongue	III	thin	Y	Y	N	Y	C	Y	apartment	N
OMM6	Mixed	F	Y	10	gingiva	III	thin	Y	N	N	N	C	Y	house	Y
OMM7	Mixed	M	Y	8	gingiva	II	obese	Y	Y	N	Y	HC	N	house	N
OMM8	Rottweiler	M	N	9	gingiva	III	obese	N	N	N	Y	HC	Y	house	N
OMM9	Boxer	M	Y	11	gingiva	III	obese	N	N	N	N	C	Y	house	Y
OMM10	Mixed	M	N	12	upper lip	II	thin	N	Y	N	Y	HC	N	house	N
OMM11	Mixed	M	Y	13	upper lip	III	adequate	N	N	N	N	HC	Y	house	Y
OMM12	Mixed	M	N	10	hard palate	III	thin	N	N	N	N	H	N	house	N
OMM13	Teckel	M	Y	14	lower lip	III	thin	Y	Y	N	Y	C	Y	apartment	N
OMM14	Poodle	M	N	10	gingiva	II	thin	Y	N	N	N	C	Y	house	Y
OMM15	Labrador	M	Y	12	gingiva	III	obese	N	N	N	Y	C	Y	house	Y
CTRL1	Mixed	M	Y	11	none	none	thin	Y	N	Y	Y	C	Y	house	Y
CTRL2	Teckel	M	N	13	none	none	thin	Y	N	N	Y	C	Y	apartment	N
CTRL3	Poodle	M	N	9	none	none	thin	N	N	N	N	C	Y	apartment	Y
CTRL4	Mixed	F	Y	13	none	none	obese	N	N	N	Y	C	N	house	Y
CTRL5	Mixed	M	Y	14	none	none	obese	Y	N	N	N	C	Y	house	N
CTRL6	Boxer	M	N	9	none	none	obese	N	Y	N	N	HC	Y	house	Y
CTRL7	Labrador	M	Y	10	none	none	obese	N	N	N	N	C	Y	house	N
CTRL8	Rottweiler	M	Y	9	none	none	obese	Y	N	N	Y	C	Y	house	Y
CTRL9	Mixed	F	Y	9	none	none	adequate	N	N	N	N	C	N	house	N
CTRL10	Mixed	F	Y	12	none	none	adequate	Y	N	N	N	C	Y	house	N
CTRL11	Mixed	F	Y	10	none	none	adequate	Y	N	N	N	C	Y	house	N
CTRL12	Cocker	F	Y	12	none	none	adequate	Y	N	N	N	HC	Y	house	Y
CTRL13	Poodle	F	Y	9	none	none	adequate	N	N	N	Y	HC	Y	apartment	N
CTRL14	Mixed	M	Y	10	none	none	adequate	Y	N	N	N	C	Y	house	Y
CTRL15	Mixed	M	Y	7	none	none	adequate	N	N	N	N	C	Y	house	N

OMM, oral mucosal melanoma; n/a, not available; F, female; M, male; Y, yes; N, no; H, homemade food; C, commercial food; HC, mixed food (homemade and commercial).

**Table 3:** Demographics of dogs enrolled into the study and comparisons between some parameters.

	*Variable*	OMM Group (15 Dogs)		Control Group (15 Dogs)		*p* Value
		*Absolute Values*	(%) Percentage	*Absolute Values*	(%) Percentage	
**Sex**	*Females*	6	40%	6	40%	0.715
	*Males*	9	60%	9	60%	
**Neutered or not**	*Not neutered*	3	20%	12	80%	0.409
	*Neutered*	5	33%	10	67%	
**Body score**	*Adequate*	3	20%	7	47%	
	*Thin*	7	47%	3	20%	
	*Obese*	5	33%	5	33%	
**Age**	*7–9 years old*	3	20%	6	40%	Not significantly different (on purpose)
	*10–12 years old*	9	60%	6	40%	
	*Above 13 years*	3	20%	3	20%	
**Breed**	*Boxer*	1	7%	1	7%	Not significantly different (on purpose)
	*Cocker Spaniel*	1	7%	1	7%	
	*Labrador Retriever*	1	7%	1	7%	
	*Poodle Standard*	2	13%	2	13%	
	*Rottweiler*	1	7%	1	7%	
	Mixed	8	53%	8	53%	
	*Teckel*	1	7%	1	7%	
**Type of nose**	Brachycephalic	1	7%	1	7%	
	Mesocephalic	11	73%	7	47%	
	Dolichocephalic	3	20%	7	47%	
**Periodontal disease**	Yes	8	53.33%	8	53.33%	Not significant
	No	7	46.70%	7	46.67%	
**Tumor localization**	Gingiva	8	53.33%	n/a	n/a	No comparisons were made
	Lower lip	1	6.67%	n/a	n/a	
	Upper lip	2	13.33%	n/a	n/a	
	tongue	1	6.67%	n/a	n/a	

**Table 4:** Demographics of animals enrolled into the study comparing environmental variables.

	*Variable*	OMM Group (15 dogs)		Control Group (15 dogs)		
		*Absolute Values*	(%) Percentage	*Absolute Values*	(%) Percentage	*p* Value
**Contact with smokers?**	No	5	33.33%	1	6.67%	0.1686
	Yes	10	66.66%	14	93.40%	
**Antiparasitary**	Yes	14	93.33%	14	93.33%	ns
	No	1	7%	1	7%	ns
**Use of disinfectants**	Yes	6	38.46%	6	39.96	ns
	No	9	58.82%	9	61.54%	ns
**Type of food**	Homemade	2	13.33%	0	0.00%	
	Commercial	9	60.00%	12	80.00%	
	Mixed (homemade and commercial)	4	26.67%	3	20.00%	
**Teeth brushing**	Yes	1	6.60%	1	6.60%	Not significantly different
	No	14	93.40%	14	93.40%	
**Dental tartar cleaning**	Yes	7	33.33%	10	66.67%	0.462
	No	8	53.33%	5	46.67%	

**Table 5:** Percentages of bacterial types in the oral cavities of OMM-bearing dogs and controls.

Bacteria Species	OMM (Mean % ± SD) (*n* = 15)	Controls (Mean % ± SD) (*n* = 15)	*p*-Value
*Tannerella forsythia*	1.71 ± 1.25	0.62 ± 0.62	0.017 *
*Porphyromonas cangingivalis* + *Tannerella forsythia*	1.71 ± 1.25	0.62 ± 0.63	0.017 *
*Bergeyella zoohelcum*	0.63 ± 1.36	4.16 ± 10.86	0.027 *
*Porphyromonas canoris*	2.33 ± 3.99	4.11 ± 4.78	0.128
*Bacteroides pyogenes*	3.45 ± 5.21	1.81 ± 4.52	0.180
*Filifactor alocis*	0.57 ± 1.17	0.8 ± 1.17	0.222
*Neisseria shayeganii*	1.95 ± 2.95	0.9 ± 1.72	0.310
*Capnocytophaga cynodegmi*	4.47 ± 15.89	0.89 ± 1.66	0.356
*Moraxella* sp.	10.36 ± 14.21	12.51 ± 16.69	0.367
*Capnocytophaga canimorsus*	1.75 ± 5.18	1.84 ± 5.22	0.433
*Wolinella succinogenes*	1.39 ± 2.21	1.27 ± 1.56	0.482
*Fusobacterium nucleatum*	5.96 ± 8.98	2.32 ± 2.46	0.549
*Porphyromonas cangingivalis*	19.15 ± 17.46	14.92 ± 15.86	0.564
*Porphyromonas gingivicanis*	2.39 ± 2.13	3.12 ± 3.09	0.580
*Pasteurella canis*	1.6 ± 2.36	0.97 ± 1.61	0.612
*Pasteurella dagmatis*	1.69 ± 2.06	4.78 ± 12.33	0.712
*Porphyromonas gulae*	13.32 ± 15.87	9.93 ± 12.84	0.729
*Treponema denticola*	0.92 ± 0.97	1.17 ± 1.32	0.818
*Conchiformibius steedae*	3.14 ± 5.43	2.85 ± 4.68	0.907
*Fusobacterium russii*	10.15 ± 12.22	9.87 ± 12.68	1.000
*Campylobacter rectus*	2.4 ± 1.54	2.47 ± 1.78	1.000

* indicates that the values are significantly different.

## Data Availability

Data are available upon request.

## Supplementary Materials

The following supporting information can be downloaded at: https://www.mdpi.com/article/10.3390/cancers14143397/s1, Supplementary Material: Epidemiological Qustionare Applied to Tutors.
