# Supplementary material for: Unraveling the Risk Factors and Etiology of the Canine Oral Mucosal Melanoma: Results of an Epidemiological Questionnaire, Oral Microbiome Analysis and Investigation of Papillomavirus Infection
Source: Cancers (Basel). 2022 Jul 13;14(14):3397. doi: 10.3390/cancers14143397 (PMC9316277; doi:10.3390/cancers14143397)
Supplement: Supplementary file 1 [file cancers-14-03397-s001.zip › cancers-1740793-supplementary.pdf]

SCHOOL OF VETERINARY MEDICINE AND ANIMAL SCIENCE OF THE UNIVERSITY OF SAO PAULO

Research project: Oral Mucosal Melanomas in dogs: is there a correlation with the environment and life style of the animal?

Laboratory of Experimental and Comparative Oncology

Department of Pathology

Coordination: Maria Lucia Zaidan Dagli

Students: Marcella Collaneri Carrilho (Scientific Initiation)

Joyce Pires de Carvalho (Master in Science student)

Name of the veterinary hospital ________________________________
Registration Number ____

EPIDEMIOLOGICAL QUESTIONNAIRE APPLIED TO TUTORS

1. Tutor Name/Phone

2. Dog's Name/Age

3. Breed

4. Address

( ) house ( ) apartment

5. ZIP CODE and neighborhood name

6. Has your dog had or has oral melanoma?
□ yes □ no

7. The dog drinks water from
□ tap □mineral □pool
□ rain ◎ lake

8. Gender of the animal
□female □female spayed
□male □neutered male

9. Weight
□lower than 10Kg □10-25 Kg. □ higher than 25 Kg

10. Your dog can be considered:
□ thin □under weight
□ normal weight □ overweight. □ obese

11. Your dog's nose is
□ long. □ medium. □ short

12. How long has the animal lived with the same owner at the same address?
____years. _____months

13. Where does the dog spend 2/3 of its time per day?
____ garden
____garage
____in a company/store
____in an industrial area
____in a rural area
____other ___________

14. Does the dog live near a busy avenue or street?
□ yes □ no

15. The dog:
□ lives most of the time indoors Indoors and in the yard
□ lives most of the time in the yard.
other locations: ______________

16. The yard has:
□floor □grass □plants
□cement □earth

17. The dog lives most of its life:
□ In urban areas □ In rural areas

18. Do you usually barbecue or burn wood (fire) in the place where the dog circulates?
___No
___once a week
___twice a week
___three times a week
___more than 3 times a week

19. Can the dog have access to building materials that contain paint, solvent, formaldehyde, asbestos or other products?
____no
____frequently
____I don't know
____ocasionally
____rarely
____yes
Which product (s)?___________

20. The dog has contact with herbicide (stored in the garden)?
____no
____frequently
____I don't know
____ocasionally
____rarely
____yes
Which product (s)?___________

21. Does the dog have contact with fungicides (to eliminate fungi, plant diseases due to fungus) that are stored or sprayed on plants in the garden or house?
____no
____frequently
____I don't know
____ocasionally
____rarely
____yes
Which product (s)?___________

22. Does the dog have contact with insecticides in the house, in cupboards, on the floor, plants or elsewhere?
____no
____frequently
____I don't know
____ocasionally
____rarely
____yes
Which product (s)?___________

23. Is there wireless (wi-fi) in the room where the dog sleeps?
□ no □yes

24. Is there a cordless phone in the room where the dog sleeps?
□ no □yes

25. Does the dog have contact with used household products or biological products for house maintenance?
____no
____frequently
____I don't know
____ocasionally
____rarely
____yes
Which product (s)?___________

26. Do you use incense, candles, fragrances, scented oils where the dog roams?
____no
____frequently
____I don't know
____ocasionally
____rarely
____yes
Which product (s)?___________

27. The dog is in the same environment as people who smoke
□cigarette □cigar □pipe

28. Does the dog have contact with people who smoke (inside the house or in other enclosed places or in the car)?
____no
____frequently
____I don't know
____ocasionally
____rarely
____yes
Which product (s)?___________

How many packs of cigarettes a day?

□<1 □1 □2 □3 □4 □5 □6 □7□8

How long have you smoked or smoked in the same place as the dog?
____ years. ____months

29. How many people smoke in the house?

□ 1 □2 □3 □4 □5 □6 □7□8

30. Do you know any dogs with oral melanoma in the neighborhood?
□ no □yes □ don't know

31. Do you use shampoo with ectoparasiticide in your dog's bath?
____no
____frequently
____I don't know
____ocasionally
____rarely
____yes
Which product (s)?___________

32. How many times a year do you treat your dog with some kind of external anti-parasite?
___ times per year
___never

33.What kind of antiparasitic do you usually use on your dog?
___none
___Commercial product name __________
___Anti flea pills

34. Do you use in the environment the following solutions?
□ amitraz □ lindane □others:

35. What is your dog's diet?
___homemade
___commercial
___mixed

36. Does the dog eat fruits or vegetables?
___yes
___no
___ I don’t know

37. Does anyone in contact with the dog work with:
□ industry (Which one?)
□ painting □ bodywork
□ construction □agriculture
□ woodworking □others:

38. Do you brush your dog's teeth?
___no
___once a day
___twice a day or more

39. Has your dog ever had a dental cleaning performed by a veterinarian? How many times?
___no
___once
___twice or more

40. Does your dog have a history of periodontal disease?
___yes
___no

41. Do you think your dog is exposed to a specific cause that could develop or have developed cancer? Why?

___

42. Location of Oral Melanoma:

- **□ Gum □ Tongue**
- **□ Hard palate □Soft palate**
- **□ Retropharyngeal space**
- **□ Upper lip**
- **□ Lower lip**

43. STAGING OF ORAL MELANOMA according to Owen, 1980*, and Bergman, 2007

□ Stage I: Tumor less than 2 cm in diameter, without involvement of regional lymph nodes or metastases.

□ Stage II: Tumor between 2 and 4 cm in diameter, without involvement of regional lymph nodes or metastases.

□ Stage III: Tumor greater than 4 cm in diameter, with or without histological or cytological evidence of regional lymph node involvement, without metastases.

□ Stage IV: Tumor of any size, with or without lymph node involvement and metastases.

References

Owen, L. N, World Health Organization. Veterinary Public Health Unit & WHO Collaborating Center for Comparative Oncology. (1980). TNM Classification of Tumours in Domestic Animals/ edited by L.N. Owen. World Health Organization. https://apps.who.int/iris/handle/10665/68618

Bergman PJ. Canine oral melanoma. Clin Tech Small Anim Pract. 2007 May;22(2):55-60. doi: 10.1053/j.ctsap.2007.03.004. PMID: 17591290.

Supplementary material

Results of the epidemiological questionnaire

Question	OMM cases (n=15)	Non-OMM cases (controls) (n=15)
Sex	6 females, 9 males	6 females, 9 males
Neutered?	10 yes, 5 no	12 yes, 3 no
Body score	5 obese, 3 adequate, 7 thin	5 obese, 7 adequate, 3 thin
Ages	7 – 9 years old –3<br>10 – 12 years old-9<br>13 yrs old or more- 3	7 – 9 years old – 6<br>10 – 12 years old-6<br>13 yrs old or more-3
Breed	7 Mongrel, 8 pure breed	8 Mongrel, 7 pure breed
Type of housing	13 house; 2 apartment	12 house; 3 apartment
Type of nose	3 long, 1 short, 11 medium	7 long, 1 short, 7 medium
Living zone	14 urban, 1 rural	14 urban, 1 rural
Exposure to smoke	1 yes, 14 no	2 yes, 13 no
Close to chemicals? Access to building materials that contain paint, solvent, formaldehyde, asbestos or other products?	1 yes, 14 no	2 yes, 13 no
Use of insecticides	5 yes, 10 no	7 yes, 8 no
Wi-fi	10 yes, 5 no	12 yes, 3 no
House cleaning products	11 yes, 4 no	11 yes, 4 no
Contact with smokers	6 yes, 9 no	1 yes, 14 no
Presence of ectoparasites	14 yes, 1 no	14 yes, 1 no
Use of antiparasitary drugs	14 yes, 1 no	14 yes, 1 no
Disinfectants	10 yes, 5 no	7 yes, 8 no
Type of food	2 homemade only, 9 commercial only, 4 mixed	0 homemade only, 12 commercial only, 3 mixed
Eat fruits?	12 yes, 4 no	13 yes, 2 no
Brush the teeth?	1 yes, 14 no	1 yes, 14 no
presence of dental tartar	8 yes, 7 no	5 yes, 10 no
Periodontal disease	7 yes, 8 no	8 yes, 7 no
Localization of the tumor	3 Palate, 8 gingiva, 3 lips, 1 tongue	Not applicable
Stage of the tumor	Stage II – 5 animals<br>Stage III – 10 animals	Not applicable
